# PEAC: An Ultrasensitive and Cost-Effective MRD Detection System in Non-small Cell Lung Cancer Using Plasma Specimen

**DOI:** 10.3389/fmed.2022.822200

**Published:** 2022-03-03

**Authors:** Jianping Xu, Yue Pu, Rui Lin, Shanshan Xiao, Yingxue Fu, Tao Wang

**Affiliations:** ^1^Department of Medical Oncology, Cancer Hospital, Chinese Academy of Medical Sciences, Beijing, China; ^2^Peking Union Medical College, Beijing, China; ^3^Department of Research and Development, Hangzhou Repugene Technology Co., Ltd., Hangzhou, China

**Keywords:** liquid biopsy, CtDNA, ddPCR, NGS, PEAC, MRD

## Abstract

Circulating tumor DNA (ctDNA), a tumor-derived fraction of cell-free DNA (cfDNA), has emerged as a promising marker in targeted therapy, immunotherapy, and minimal residual disease (MRD) monitoring in postsurgical patients. However, ctDNA level in early-stage cancers and postsurgical patients is very low, which posed many technical challenges to improve the detection rate and sensitivity, especially in the clinical practice of MRD detection. These challenges usually include insufficient DNA input amount, limit of detection (LOD), and high experimental costs. To resolve these challenges, we developed an ultrasensitive ctDNA MRD detection system in this study, namely PErsonalized Analysis of Cancer (PEAC), to simultaneously detect up to 37 mutations, which account for 70–80% non-small cell lung cancer (NSCLC) driver mutations from low plasma sample volume and enables LOD of 0.01% at a single-site level. We demonstrated the high performance achieved by PEAC on both cfDNA reference standards and clinical plasma samples from three NSCLC patient cohorts. For cfDNA reference standards, PEAC achieved a specificity of 99% and a sensitivity of 87% for the mutations at 0.01% allele fraction. In the second cohort, PEAC showed 100% concordance rate between ddPCR and Next-generation sequencing (NGS) among 29 samples. In the third cohort, 22 of 59 patients received EGFR TKI treatment. Among them, three in four patients identified low level actionable gene mutations only by PEAC had partial responses after targeted therapy, demonstrating high ctDNA detection ability of PEAC. Overall, the developed PEAC system can detect the majority of NSCLC driver mutations using 8–10 ml plasma samples, and has the advantages of high detection sensitivity and lower costs compared with the existing technologies such as ddPCR and NGS. These advantages make the PEAC system quite appropriate for ctDNA and MRD detection in early-stage NSCLC and postsurgical recurrence monitoring.

## Introduction

As an extensively studied liquid biopsy method, cell-free circulating tumor DNA (ctDNA) from plasma samples has broad clinical applications in targeted therapy (1), immunotherapy (2), and perioperative minimal residual disease (MRD) monitoring (3) for cancer patients. ctDNA liquid biopsy has several advantages including sample availability, non-invasiveness, and lower intratumor heterogeneity. ctDNA assay has been widely used in patients with established metastatic disease to mostly identify therapeutically actionable tumor mutations. Recently, highly sensitive testing approaches that enable reliable detection of low abundant ctDNA show great potential in MRD detection, which complement CT imaging for tumor recurrence surveillance after surgery (3–5). Early detection of micrometastatic lesions might lead to better post-adjuvant intervention before tumor recurrence. However, ctDNA level varies greatly in different cancer types and cancer stages. To effectively detect MRD, the limit of detection (LOD) of mutant allele fraction should be lower than 0.1% (6). In addition, periodically dynamic ctDNA detection might also provide early surrogate endpoints for clinical outcomes and decrease the follow-up times and costs for patients undergoing adjuvant therapy (3). Therefore, biomarkers derived from ctDNA showed great potential in precision treatment, MRD monitoring of tumor recurrence, etc. (7).

Lung cancer is the most commonly diagnosed malignant tumor and is a leading cause of cancer-related death worldwide (8, 9). Somatic mutations which frequently occur in EGFR tyrosine kinase domain may predict response to EGFR tyrosine kinase inhibitors (TKIs) in patients with non-small cell lung cancer (NSCLC). Among EGFR activating mutations, 19del and L858R were found in 45–55% east Asian patients with NSCLC, while rare EGFR mutations including S768I, L861Q, G719X, etc. have the prevalence of 1.1–2.4%, respectively (10). Driver mutations from downstream MAPK pathway, such as KRAS G12X/G13X, was previously considered an “undruggable” target and one that would cause EGFR-TKI resistance, but now it showed impressive drug efficacy in the clinical trial (11). Moreover, BRAF V600E, ALK/RET/ROS1 gene fusions, PIK3CA, and NRAS mutations are also therapeutically actionable mutations and they are either sensitive or resistant to specific TKIs (12, 13). Besides acting as TKI therapy targets, those driver mutations largely belong to the clone mutations, and might reappear in cancer relapse (14). Thus, there is a great need to simultaneously detect those therapeutically actionable or driver mutations by ctDNA liquid biopsy in both circumstances when tissue specimens cannot be obtained repeatedly (15).

To accurately detect low-level ctDNA and MRD, we thoroughly investigated available ctDNA detection technologies, such as widely used Amplification Refractory Mutation System (ARMS), digital PCR (dPCR)/digital droplet PCR (ddPCR) (16, 17), Next-generation sequencing (NGS) technology (18, 19), and several blocker sequence-based PCR enrichment methods (20–38). ARMS–PCR is clinically affordable, but it can only detect one mutation per assay and has a limit of detection ranging between ~0.1 and 1% using 4 ml plasma (39, 40). dPCR showed high sensitivity and absolute quantification by partitioning the sample into thousands of single molecular compartments and could achieve a detection limit of ~0.01% (41). Though highly sensitive, it requires lots of assay optimization and has limited multiplexing capacity to simultaneously detect several mutations per assay. In practice, ddPCR assay can only detect 2–3 known mutations using 8–10 ml plasma (42–44). NGS technology has the advantages of high throughput and the ability to detect almost all kinds of mutation types, such as SNV, indel, fusion, and copy number variation (CNV), but it usually has a complicated workflow and detection limit of 0.1% due to sequencing artifacts and PCR errors (45). In the TRACERx study (6, 46), a personalized UMI-based multiplex PCR NGS approach was developed to robustly detect MRD at a median mutant allele fraction of <0.1% (6, 46, 47). CAPP-Seq, another ultrasensitive NGS method, can achieve 96% specificity for mutant allele fractions down to ~0.02% (48) and be applied to MRD detection in localized lung cancer after treatment (47). Although optimized to achieve high sensitivity, these NGS technologies require high sequencing depth (>10,000X) and is costly during periodical MRD testing. In consideration of both detection limits and accumulated MRD testing costs, we reviewed other simple, sensitive, and cost-effective technologies, which include peptide nucleic acid (PNA)-based method (23, 29, 34, 36), locked nucleic acid (LNA)-based method (21, 22, 25, 26, 28, 31, 32), dual priming oligonucleotide (DPO)-based method (20, 24, 27, 30, 33, 35, 37), and blocker displacement amplification (BDA) (38). Except for BDA, the mentioned approaches used modified blocker sequence with outperformed affinity to bind with the complementary sequence other than the sequence with a certain mutation. However, it has been challengeable to scale these approaches to multiplexed enrichment of many different variants because existing methods require that the operational reaction temperature sits in the “Goldilocks” zone of every single blocker. BDA was firstly reported by Lucia R. Wu which was based on the toehold-exchange mechanism using a rationally designed competitive hybridization reaction. The competitive hybridization reaction enables PCR not only to sensitively recognize and selectively amplify SNVs, but also to do so across a broad temperature window spanning 8°C, thus conveniently accomplishing multiplexing purposes. Several BDA-derived methods showed high sensitivity to detect low-abundant ctDNA mutations with cost-effective experimental procedures (49–52). Therefore, we believe that these kind of technologies are quite appropriate to implement a highly sensitive, multiplexing-enabled, and cost-effective MRD detection system for NSCLC. However, we found that BDA has some technical problems: (1) It might introduce additional base substitutions under some enrichment conditions, thus creating false positives. (2) Constructing a multiplexing system is not a trivial task and there need many considerations of primer, blocker designs, and systematic validation of the pooled mutation assay system.

In this study, we improved BDA technology and described an ultrasensitive and cost-effective variant detection system, namely PErsonalized Analysis of Cancer (PEAC), to detect the majority of the NSCLC driver mutations using ctDNA plasma samples. The principle of this system combined the above-mentioned BDA and false-positive eliminating methods and utilized differential binding ability between the primers and the blockers to specifically enrich mutant DNA fragments, and then sequenced the enriched products by either Sanger sequencing or NGS sequencing platform. We demonstrated that PEAC can enriched 37 mutations with the limit of detection of 0.01% in two reliable reaction assays, which cover 70–80% of therapeutically actionable mutations in NSCLC (10). The performance of the PEAC system was validated, and the features of high sensitivity and cost savings made it quite appropriate for the clinical applications such as ctDNA detection in early-stage NSCLC and repeated postsurgical MRD monitoring.

## Materials and Methods

### The Principle of PEAC System

**Figure 1:**
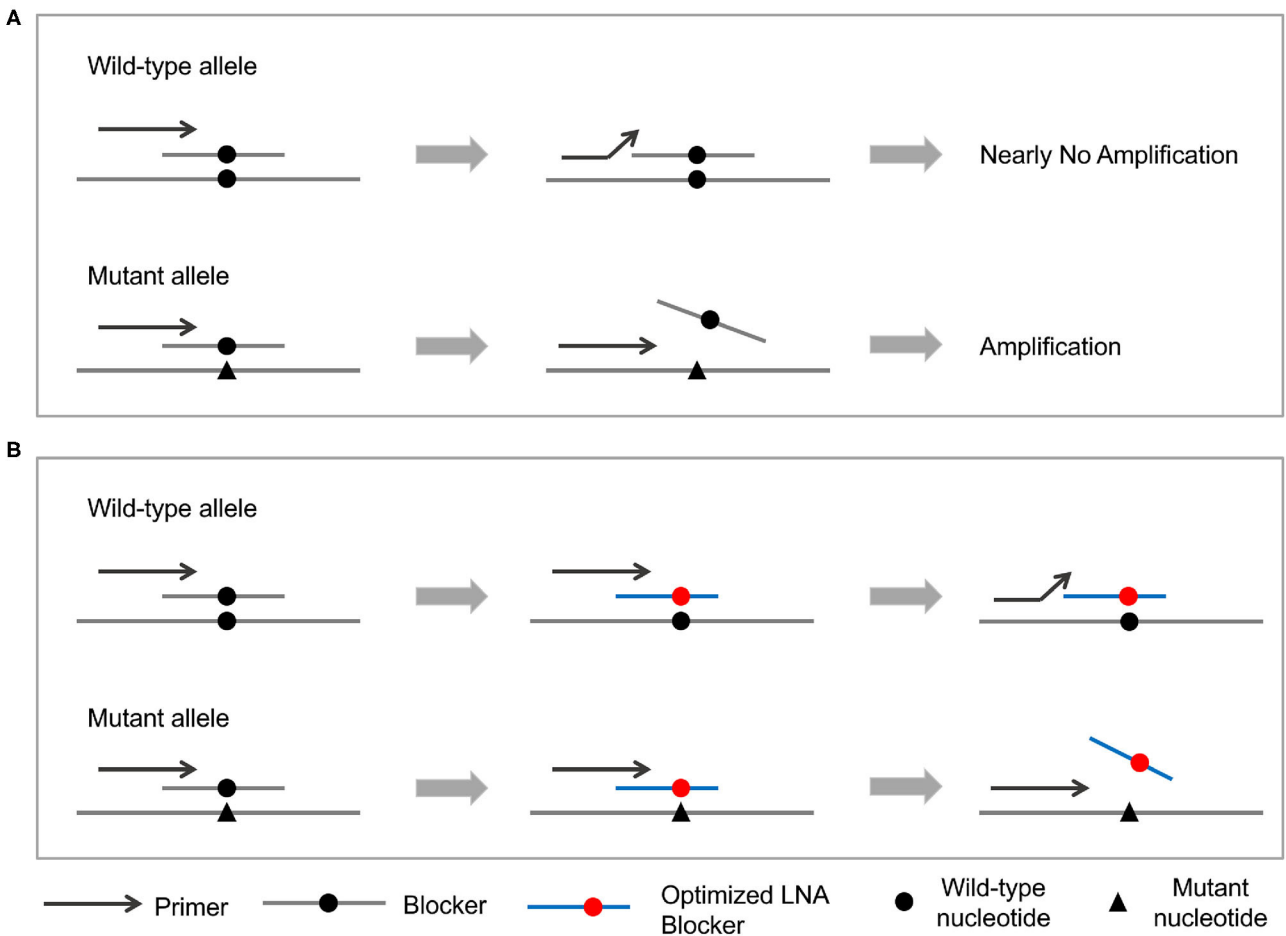
Principle of the PEAC methodology. **(A)** The general PEAC workflow for NSCLC driver mutations. Forward primers and blocker probes are competitively binding to the DNA template. For wild-type template, blocker probes prevent primers binding and cause no PCR amplification. On the other hand, blocker probes could not bind to the mutant template and let the PCR amplification goes unhindered. **(B)** Adjustment of PEAC method for specific mutations, such as EGFR T790M and L861Q. These two blocker probes are modified with locked-nucleic acid (LNA) and with optimized length eliminate the false positives.

Personalized analysis of cancer system achieves the enrichment of PCR products of mutant types by creating a differential binding ability between primers and blocker probes (Figure 1). More specifically, primers have a stronger binding affinity than blocker probes for mutant templates, and so the PCR amplification goes unhindered. On the other hand, blocker probes prevent primers extension for wild-type templates. These differential amplification characteristics would be extremely amplified through multiple cycles of PCR and enable extremely high sensitivity. Some of these blocker probes were modified with locked-nucleic acid (LNA) (53–55) and with optimized length to solve the problem of false positive.

The mutation on the mutant template will be enriched if it is located in the region of non-overlap part of the blockers, which we called enrichment region. The PEAC system was designed for 10 enrichment regions, which cover EGFR exons 18–21 and enable to detect mutations including EGFR L858R, EGFR T790M, EGFR 19DEL, etc. (Supplementary Table 1). These 10 enrichment regions were experimentally grouped into two reaction assays. One reaction detects the mutations including EGFR L858R, EGFR T790M, EGFR 19DEL, KRAS G12D, NRAS Q61K, PIK3CA E545K, etc., and the other reaction detects EGFR L861Q, EGFR G719S, EGFR S768I, BRAF V600E, etc. The whole system has a multiplexing mutation detection capacity of 37 mutations currently.

### Design of Primers and Blockers

There exist several design concepts of primers and blockers. Firstly, forward primers (also called enrichment primers) should be placed close enough to the position of the mutant locus to be detected, which would be covered by blockers and having a Tm value between 57 and 60°C. Secondly, blockers should perfectly match the wild-type template and they had a Tm value between 60 and 64°C. For the modified blocker, mutant locus is modified by LNA and the Tm value of the modified blocker is the same around 60–64°C. Thirdly, the 3' end of the blocker is modified with a phosphate group to prevent its amplification during PCR. Fourthly, the length of the overlap part between the primers and blockers should be 6–14 bases. The Tm value of the non-overlap part of the blocker is generally higher than that of the non-overlap part of the enrichment primer. Lastly, reverse primers were designed to avoid dimers with enrichment primers and blockers. In total, we designed ten pairs of primers and ten blockers (Supplementary Table 2) for the PEAC system.

### CfDNA Reference Standards on Various Allele Fraction Levels

We generated cfDNA reference standards (Horizon Discovery, Cambridgeshire, UK) for the 37 mutations at five different allele fraction levels (1, 0.1, 0.05, 0.01%, and wildtype). Six of these mutations, i.e., EGFR L858R (c. 2573T>G), T790M (c.2369G>A), E746_A750del (c. 2235_2249 del15; named as 19del hereafter), KRAS G12D (c.35G>A), NRAS Q61K (c.181C>A), and PIK3CA E545K (c.1633G>A) mutations, were included in the first mutation set at 1, 0.1%, and wildtype allele fraction levels. We then generated 0.05 and 0.01% mutations by mixing the 0.1% and wildtype reference standards. The cfDNA standards of five allele fraction levels for the other four mutations, including EGFR L861Q, G719S, S768I, and BRAF V600E, were made following the same procedure.

### PEAC Enrichment Followed by Sanger

For each mutation enrichment reaction, to obtain a final concentration of 200 nM of forward primer-1 and reverse primer-1, the corresponding amount of blockers for each enrichment region (Supplementary Table 2) and 20–60 ng DNA samples were mixed together. The first round of PCR reaction was performed in 100 μL reaction volume with Platinum™ SuperFi II PCR Master Mix (Thermo Fisher Scientific, Waltham, MA, USA). PCR was initially denatured at 98°C for 3 min, followed by 37 cycles of 98°C for 10 s, 60°C for 30 s, and then finished at a final extension of 72°C for 3 min. Next, 5 μL of the first-round PCR products was used for the second round PCR reaction for each enrichment region by using the final concentration of 400 nM paired forward primers and reverse primers (Supplementary Table 3) and Phusion® High-Fidelity PCR Master Mix with GC Buffer (NEB, M0532L). The PCR conditions were 98°C: 3 min, (98°C: 10 s, 60°C: 30 s) × 30), 72°C: 3 min, and stored at 4°C. Mutation status of DNA fragments after PEAC enrichment can be easily examined by electrophoresis, and then sequenced using ABI 3730XL DNA Analyzer according to the manufacturer's instructions (Applied Biosystems, Foster City, CA, USA). Chromatograms were analyzed using SnapGene software version 3.2.1 (GSL Biotech LLC, Chicago, IL, USA). The enriched mutant allele fraction can be read out through their peak heights using standard Sanger sequencing chromatograms.

Before mixing the mutations into two reactions, we first found the optimized primers and blockers for each mutation. The designed primers and blockers are shown in Supplementary Table 2. We used previously prepared reference standards and 20 biological replicates to assess the PEAC performance. The DNA start amount was set differently for each reference standard, that is, 20 ng for cfDNA reference standards of 1 and 0.1% VAFs and wildtype, 40 ng for cfDNA 0.05% standards, and 60 ng for cfDNA 0.01% standards, respectively. Although most of the clinical samples have median cfDNA amount between 20 and 40 ng from 4 ml plasma, theoretically it only contains 1.8 mutant copies for 60 ng DNA at 0.01% allele fraction. Thus, we used 60 ng cfDNA at 0.01% to evaluate the limit of detection of the PEAC system.

### PEAC Enrichment Followed by NGS

Next-generation sequencing after PEAC system enrichment provides another unified and efficient way to read out the pooled 37 mutation status in one sequencing run with only a few sequencing reads. The workflow of PEAC system enrichment followed by NGS is largely the same as that followed by Sanger, but contains several modifications in PCR cycles, primers, and reaction components in the second round PCR. Specifically, the PCR cycles are changed to 20 and 25 in the first and second round PCR, respectively. PCR primers (Supplementary Table 4) and commercial NGS index primer pairs are added into the second round PCR. Compared to the Sanger workflow which needs 10 reactions in the second PCR, NGS workflow only needs two PCR reactions. After two rounds of PCR, the products were purified using the Agencourt Ampure beads following the recommended protocol for NGS sequencing. The library concentration and fragment size were evaluated with Qubit 3.0 Fluorometer (Invitrogen™; Thermo Fisher Scientific, Waltham, MA, USA) and 2100 Bioanalyzer (Agilent Technologies, Palo Alto, CA), respectively. The prepared NGS libraries were sequenced on Illumina HiSeq X Ten platform (San Diego, CA, USA) with 100 MB sequencing data yield. Raw sequencing data were demultiplexed, converted to FASTQ, mapped onto hg38, and stored in BAM files. Variant calling was performed using Vardict (version: 1.6.0).

### Clinical Validation

#### Patient Sample Collection and DNA Extraction

To validate the performance of PEAC system, we retrospectively collected three cohorts of patients with NSCLC. The first cohort was used to compare the concordance rate among PEAC system, ddPCR, and NGS technologies which had 29 stage III/IV patients with NSCLC. The second cohort contains 76 patients which was designed to validate the detection capability of PEAC on plasma samples compared to the results of paired tissue sample by NGS from various stages in (IA/IB: 51, II: 10, III/IV: 15) patients with NSCLC. The third cohort includes 59 stage III/IV patients with NSCLC tested by ddPCR and PEAC independently, and 22 patients harboring EGFR sensitive mutation received treatment of a first-generation EGFR TKI icotinib. The clinical outcomes of the third cohort were assessed by the objective response rate (ORR) using solid tumor criteria RECIST V1.1. Informed consent forms were obtained from all patients and it was approved by the Ethics Committee of Cancer Hospital, Chinese Academy of Medical Sciences & Peking Union Medical College. cfDNA was extracted from 4 mL plasma in the first and third cohorts, while 8 mL plasma in the second cohort using QIAamp Circulating Nucleic Acid Kit (Qiagen, Hilden, Germany) following the manufacturers' protocols. Genomic DNA was extracted from tissue using the QIAamp DNA FFPE Tissue kit (Qiagen, Valencia, CA, USA) and quantified using Qubit 3.0 Fluorometer and Qubit dsDNA HS Assay kit (Invitrogen, Carlsbad, CA, USA) according to the manufactures' instructions.

#### Droplet Digital PCR

For dPCR verification, 20 ng of each DNA sample from patients with NSCLC were analyzed using a QX200 Droplet Digital PCR System (Bio-Rad, Pleasanton, CA, USA). The primers, WT and mutant Taqman probes, DNA sample, and ddPCR Supermix for Probes (Bio-Rad) were mixed and loaded onto the DG8 cartridges (Bio-Rad), and the droplets were generated using the QX200 Droplet Generator (Bio-Rad) and Droplet Generation Oil for Probes (Bio-Rad). Thermal cycling was 95°C for 10 min– (94°C:30s−60°C:1 min) × 40–98°C: 10 min−4°C: hold. After PCR, the plate was loaded onto the QX200 Droplet Reader (Bio-Rad) and the droplet fluorescence data was generated. NSCLC driver mutations including EGFR L858R, 19del, T790M etc. were detected by ddPCR for the 29 plasma samples in the first cohort.

## Results

### PEAC Enrichment Followed by Sanger

**Figure 2:**
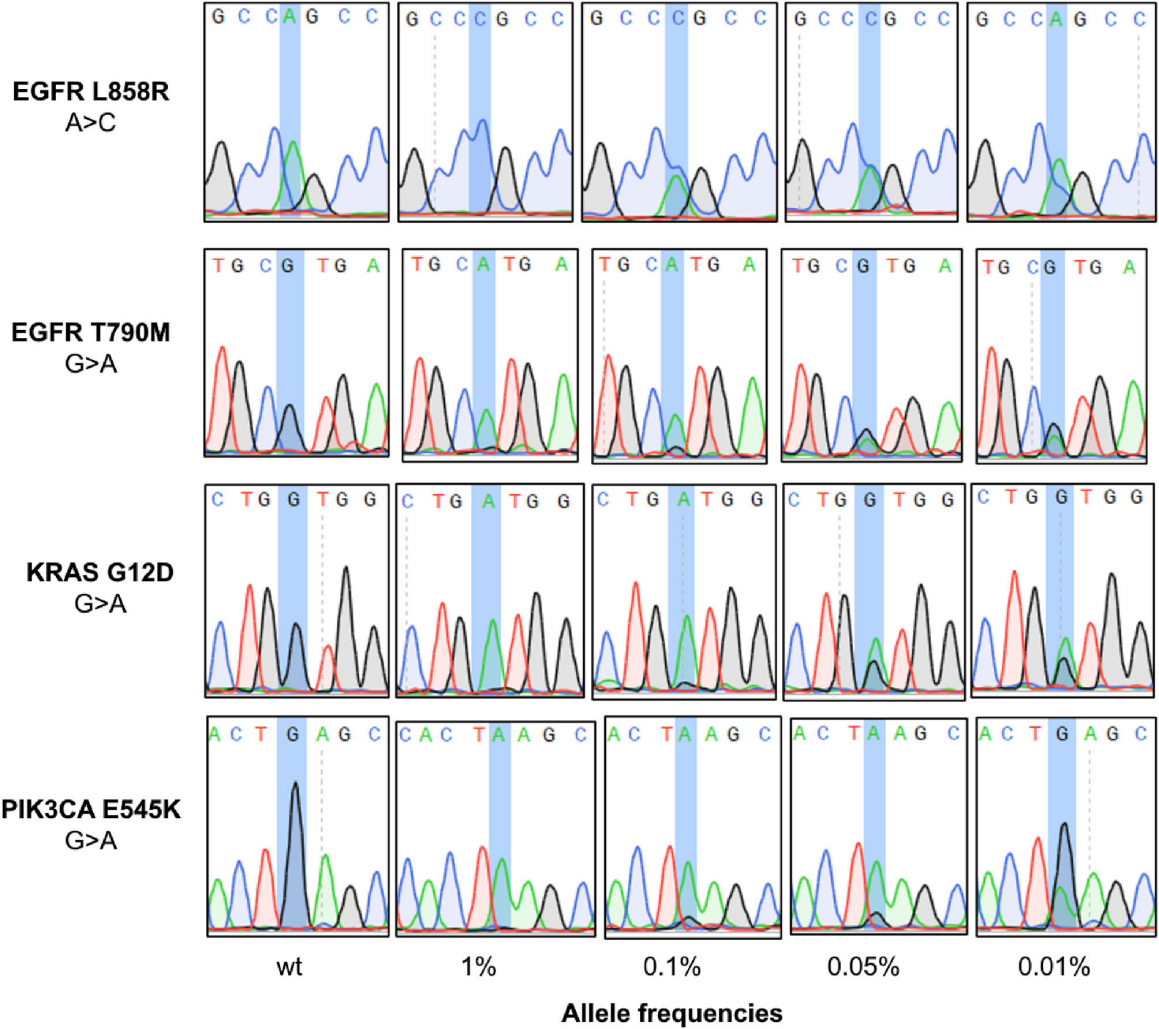
Application of PEAC enrichment followed by Sanger sequencing on NSCLC driver mutations. The mutation status can be determined by read out of the Sanger chromatograph. From the top to the bottom panels are the status of WT, 1, 0.1, 0.0%, and 0.01% reference standards after PEAC enrichment for EGFR L858R, EGFR T790M, KRAS G12D, and PIK3CA E545K mutation.

After PEAC enrichment, the PCR products were sequenced by Sanger to examine the mutation status. The wildtype mutation status can be determined by read out of the Sanger chromatograph if only the wildtype peak was shown there (Figure 2). Mutant allele of cfDNA standards at VAFs from 0.01 to 1% can be readout through the peak representing the corresponding mutations in the Sanger chromatograph (Figure 2, Supplementary Figures 1, 2). We showed an example of *KRAS* G12D (G > A) mutation. Peak of reference allele “G” can be readout for the 0.05 and 0.01% reference standards, whereas peak of mutant allele “A” can also be observed at the same locus, demonstrating the feasibility of PEAC system to distinguish mutant sample from wild-type sample.

**Figure 3:**
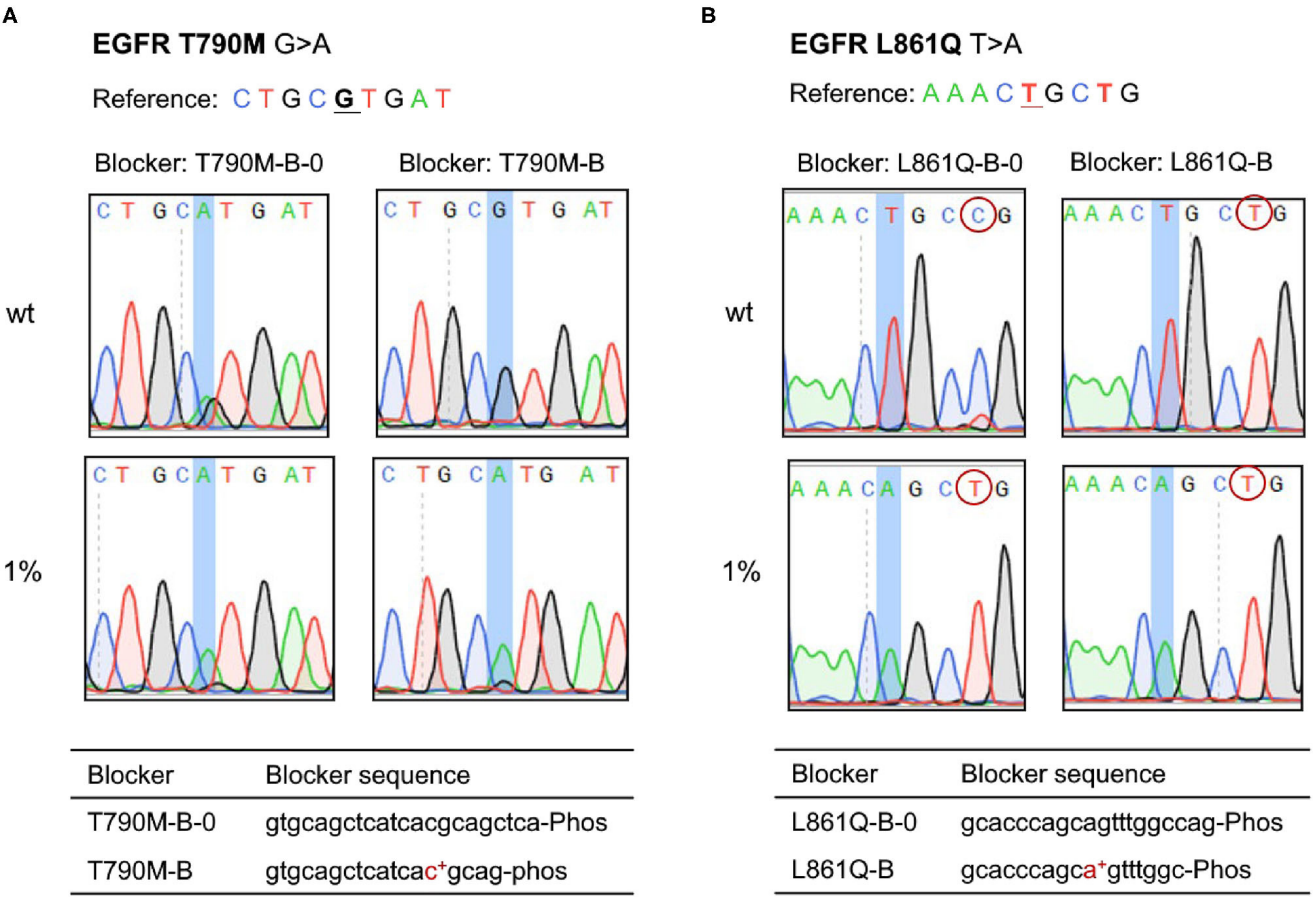
Adjustment of PEAC system for specific mutations, of which the blocker probes are modified by shortening its sequence and adding LNA-modification at the mutation site. **(A)** EGFR T790M. For T790M-B-0 probe, an additional G base was introduced within the location of T790M, leading to false positives, whereas the improved T790M-B probe solved the problem. **(B)** EGFR L861Q. For EGFR L861Q mutation reaction, when enrichment is performed using L861Q-B-0 probe, a false positive mutation of T > C shows in wild-type sample, but it is eliminated when the improved L861Q-B probe is used.

We developed the PEAC system and found it could introduce extra mutations in the enrichment region or exactly at the mutation site for EGFR T790M and L861Q reactions, thus causing false positives. To address this problem, we modified the blocker by shortening its sequence and adding LNA-modification at the mutation site (Figure 3). The improved blocker with perfect match has a similar binding strength, and overall demonstrated a greater discriminate ability between mutant and wild type templates. With such design, no additional SNV mutations were introduced in the enriched region under the same experimental conditions. Specifically, for *EGFR* T790M mutation reaction, when T790M-B-0 probe was used, an additional G was introduced within the location of T790M, leading to false positives, whereas when the improved T790M-B probe was used, the false-positive disappeared in wild type sample (Figure 3A). Similarly, for *EGFR* L861Q mutation reaction, when enrichment was performed using L861Q-B-0 probe, a false positive mutation of T > C showed in wild-type sample, but it was eliminated when the improved L861Q-B probe was used (Figure 3B). Similar results were achieved when compared G719X-B with G719X-B-0 probe.

The experiment system adopted by PEAC could enrich SNVs within the region covered by blocker sequence. Although KRAS G12D was our primary goal, we easily scaled the system to detect other mutations at the KRAS G12 and G13 locations, such as G12A, G12C, G12V, G13D, etc. (Supplementary Figure 3). The results showed that the PEAC enrichment followed by Sanger sequencing achieved superb detection capability on these mutations as well.

### Analytical Validation of PEAC Using Reference Standards

To assess the analytical performance of PEAC system, we performed validation on 20 replicates for each mutation at different mutant allele fractions of 1, 0.1, 0.05, 0.01%, and wild-type. The detection specificity and sensitivity were computed for each mutation in the PEAC system, and the results of performance are shown in Table 1. Nine of the ten mutations achieved 100% specificity except for EGFR T790M, which acquired a 90% specificity after optimizing blocker length and LNA modification. Considering only 0% specificity of T790M was achieved before optimization, such optimization played an important role in the PEAC system. The overall specificity of PEAC system was 99%. For mutation allele fractions at 1, 0.1, and 0.05%, PEAC demonstrated 100% sensitivity, thus showing the LOD of 0.05%. For ultralow allele fraction, e.g., 0.01%, PEAC system showed sensitivity that ranged between 80 and 95% for each mutation (Table 1). The false negatives are very likely because there were only several copies at the allele fraction of 0.01%, and some DNA templates were lost during DNA preparation.

**Table 1:** The specificity and sensitivity of PEAC enrichment followed by Sanger sequencing for the ten mutations in cfDNA reference standards.

**Mutations**	**Specificity**	**Sensitivity**			
		**VAF = 1%**	**VAF = 0.1%**	**VAF = 0.05%**	**VAF = 0.01%**
EGFR L858R	(20/20) 100%	(20/20) 100%	(20/20) 100%	(20/20) 100%	(18/20) 90%
EGFR T790M	(18/20) 90%	(20/20) 100%	(20/20) 100%	(20/20) 100%	(16/20) 80%
EGFR 19DEL	(20/20) 100%	(20/20) 100%	(20/20) 100%	(20/20) 100%	(19/20) 95%
KRAS G12D	(20/20) 100%	(20/20) 100%	(20/20) 100%	(20/20) 100%	(18/20) 90%
NRAS Q61K	(20/20) 100%	(20/20) 100%	(20/20) 100%	(20/20) 100%	(16/20) 80%
PIK3CA E545K	(20/20) 100%	(20/20) 100%	(20/20) 100%	(20/20) 100%	(16/20) 80%
EGFR L861Q	(20/20) 100%	(20/20) 100%	(20/20) 100%	(20/20) 100%	(18/20) 90%
EGFR G719S	(20/20) 100%	(20/20) 100%	(20/20) 100%	(20/20) 100%	(18/20) 90%
EGFR S768I	(20/20) 100%	(20/20) 100%	(20/20) 100%	(20/20) 100%	(18/20) 90%
BRAF V600E	(20/20) 100%	(20/20) 100%	(20/20) 100%	(20/20) 100%	(17/20) 85%
Total	(198/200) 99%	(200/200) 100%	(200/200) 100%	(200/200) 100%	(174/200) 87%

### Clinical Validation of PEAC Using Plasma Samples From NSCLC Patients

**Figure 4:**
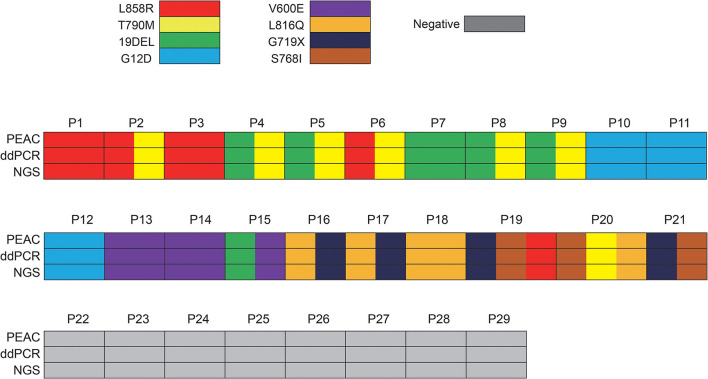
Demographics and comparison of 29 patients who received ctDNA liquid biopsy by ddPCR, NGS and PEAC following Sanger. Of these 29 patients, 21 patients harbored 8 NSCLC driver mutations including EGFR L858R, T790M, 19DEL, S768I, L861Q and G719X, KRAS G12D, and BRAF V600E, 8 patients had no mutations detected. Some patients have more than one mutation detected. The concordance rate among PEAC, ddPCR, and NGS was 100%.

To validate the performance of PEAC on clinical samples (Supplementary Table 5), we retrospectively evaluated PEAC, ddPCR, and NGS technologies on samples from 29 patients with NSCLC. NGS results showed that the 29 patients had 8 NSCLC driver mutations including *EGFR* L858R, T790M, 19DEL, S768I, L861Q, and G719X, *KRAS* G12D, and *BRAF* V600E. The variant allele fractions detected by NGS ranged between 1.25 and 48.73%. The detailed VAFs of mutations in each patient are shown in Supplementary Table 6. Comparison of the performance of the three methods showed that the mutation detection concordance rate among them was 100% (Figure 4). These results demonstrated that PEAC could stably detect those mutations in ctDNA plasma specimen from patients with NSCLC.

To investigate the detection rate in plasma samples using PEAC following Sanger sequencing, we obtained paired tissue and blood samples in the second cohort from 76 NSCLC patients. We did not apply ddPCR to this cohort due to insufficient plasma volume. Most of the patients in this cohort were stage IA/IB (67.1%, 51), which were at the early stage of cancer usually having quite low ctDNA. The remaining patients were at stage II (13.2%, 10), stage III (7.9%, 6), and stage IV (11.8%, 9), respectively. The NGS testing for tissue samples identified mutations of *EGFR* 19DEL, 20INS, G719A, G719C, L858R, L861Q, S768I, *KRAS* G12A, G12C, G12D, G12V, Q61H, *NRAS* G12D, and *PIK3CA* E545K. The allele fractions of those mutations ranged between 1.26 and 85.62% (Supplementary Table 7). We observed a high concordance rate (86.67%) between the tissue and plasma samples by PEAC in the late stage (stage III and IV) in patients with NSCLC (Table 2). It is worthy to point out that PEAC has a concordance rate of 41% between plasma and tissue samples in the early stage (stage I/II) patients, while early stage concordance rate is usually lower as shown in the previous studies (56, 57).

**Table 2:** The concordance rate of PEAC system on paired tissue and plasma samples from 76 NSCLC patients.

**Stages**	**Mutation**	**Tissue** **(NGS)**	**Blood** **(PEAC + Sanger)**	**Mutations detected in blood**	**Concordance**
Stage I and II	Positive	50	14	•EGFR L858R (6/14) •EGFR 19DEL (3/16) •Others (5/20)	41.00%
	Negative	11	11	/	
Stage III and IV	Positive	9	7	•EGFR 19DEL (3/4) •Others (4/5)	86.67%
	Negative	6	6	/	
Total	Positive	59	21	•EGFR L858R (6/14) •EGFR 19DEL (6/20) •Others (9/25)	50.00%
	Negative	17	17	/	

We further evaluated whether PEAC results on plasma samples could benefit ctDNA-guided targeted therapy. We retrospectively examined the mutation status of EGFR L858R and 19Del in the plasma samples from a third cohort. In this cohort, all patients had been tested by ddPCR and PEAC. Among them, 16 patients had EGFR L858R or 19Del mutations detected both by PEAC and ddPCR. PEAC detected an additional L858R/19del mutations in six patients and ddPCR only detected one more. As expected, these mutations only detected by one method had very low allele fractions, below 0.05%. Twenty-two L858R/19del positive patients (detected by either PEAC or ddPCR) received icotinib treatment and achieved an overall ORR rate of 59.09% (95% CI, 38.69–76.79%). Among four RECIST V1.1 evaluated patients only detected by PEAC, three patients had partial responses (PR) and one patient had stable disease (SD). That means the patients with sensitive mutations detected by PEAC at very low allele fraction might still benefit from the EGFR TKI treatment, and further demonstrate the high detection sensitivity of PEAC.

### A Case of MRD Monitoring by PEAC

**Figure 5:**
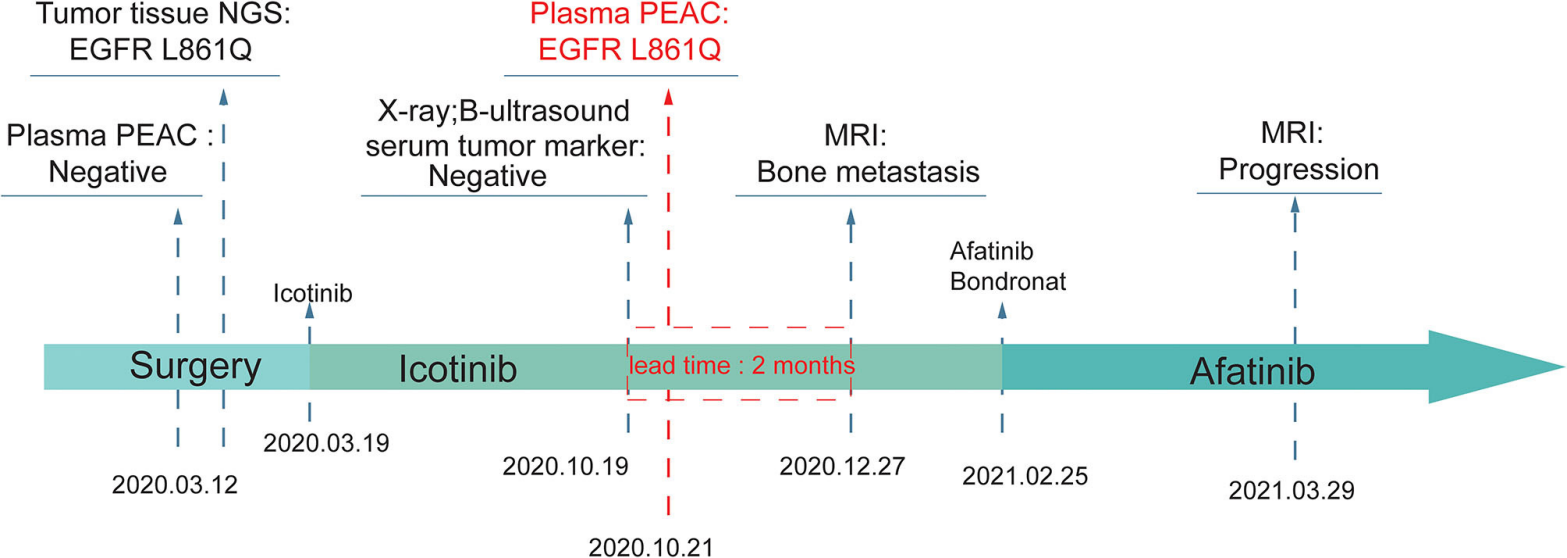
An example of MRD detection for tumor relapse monitoring using PEAC system. An EGFR L861Q mutation was detected by PEAC using plasma sample 7 months after surgery, which was also identified by NGS in tumor tissue sample. The patient was found relapsed 2 months later by magnetic resonance (MR) examination.

A 63 year old Chinese male was diagnosed with poorly differentiated adenocarcinoma at the superior lobe of his left lung with the TNM stage of T2N0M0 IB and received a series of targeted therapies (Figure 5). The patient firstly received tumor excision surgery and then a TKI-sensitive mutation, EGFR L861Q, was detected from tissue sample by a 500-gene NGS panel. The blood sample collected before surgery did not detect actionable mutation by PEAC. This patient was then treated with icotinib. After 7 months, the patient received routine X-ray, B-ultrasound, and serum tumor marker tests but showed no signs of recurrence. However, PEAC detected EGFR L861Q mutation using the plasma sample. Two months later, the patient was found to have multiple bone metastases by the magnetic resonance (MR) examination. In the clinical settings, patients usually receive a series of examinations to confirm whether disease relapsed, and important clinical information may get lost if inadequate examination happens. In such cases, PEAC can provide not only earlier cancer recurrence information but also in a simple and convenient way. This observational case demonstrated the potential benefits of MRD monitoring by PEAC for postsurgical patients.

### PEAC Enrichment Followed by NGS

Besides Sanger sequencing, NGS sequencing can also be incorporated into PEAC system. This workflow has the advantage of easy readout of all the 37 mutations and possibly can be automated in the future. A problem of sequencing uniformity among all the mutations was addressed by adjusting the input amount of forward and reverse NGS primers for the mutations in the second round PCR. As shown in Supplementary Figure 4, by applying PEAC enrichment followed by NGS to the cfDNA reference standards, the sequencing depths of those mutations were well-balanced. The optimized primer amount was further validated by 10 biological replicates and showed quite consistent results.

**Figure 6:**
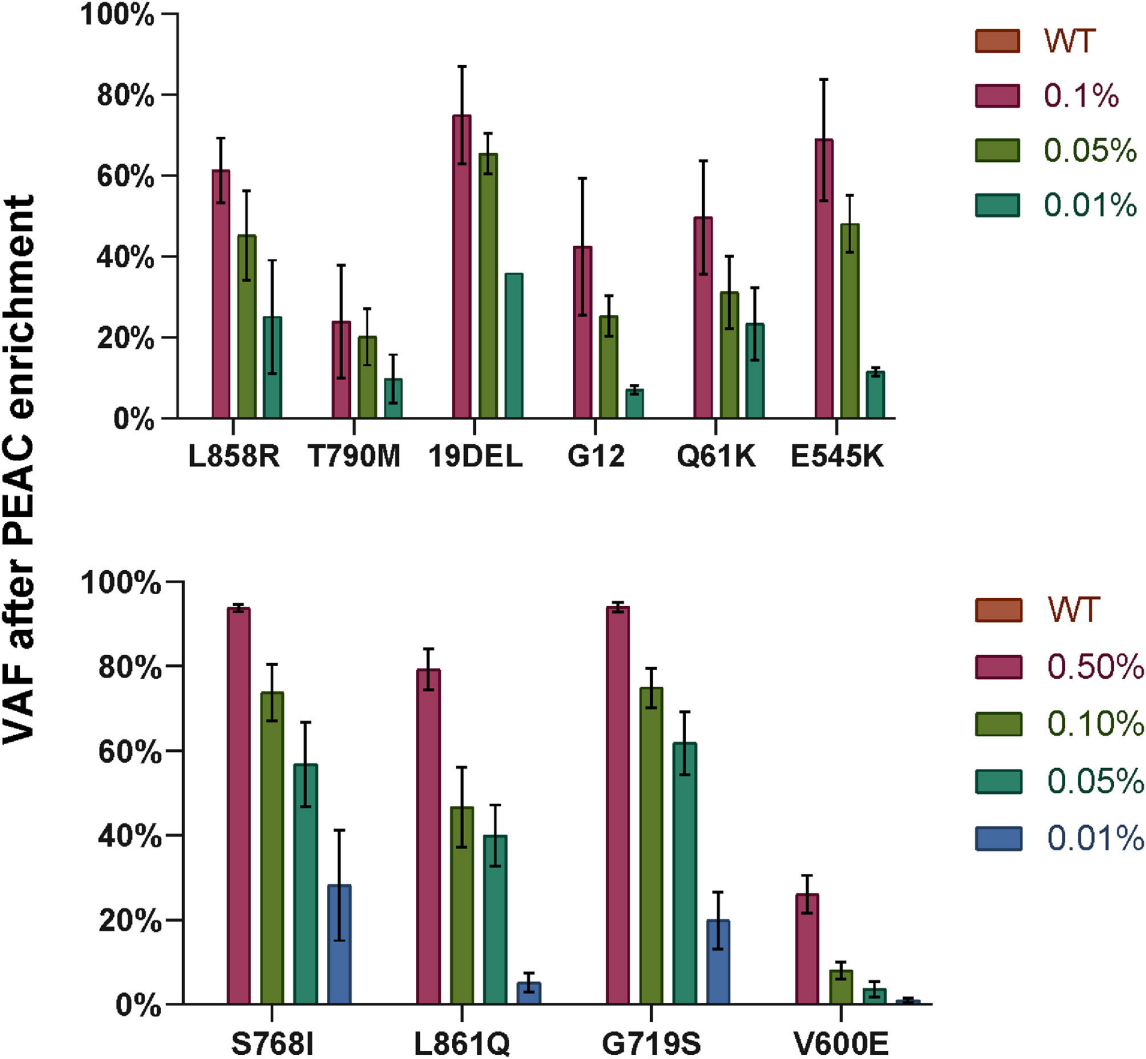
The variant allele fractions (VAFs) of the mutations detected by NGS after PEAC enrichment on cfDNA standards at different allele fractions. Ten enrichment reactions were grouped into two PCR reactions, top part showed EGFR L858R, EGFR T790M, EGFR 19DEL, KRAS G12, NRAS Q61K, and PIK3CA E545K, and bottom part included EGFR S768I, EGFR L861Q, EGFR G719S, and BRAF V600E. The bar heights represent the VAF detected by NGS after PEAC enrichment.

We then examined the feasibility of PEAC followed by NGS on cfDNA standards of the 37 mutations at 0.5, 0.1, 0.05, 0.01% VAFs, and wild-type (Figure 6). The DNA input amount was set as follows: 20 ng for cfDNA wildtype, 0.5 and 0.1%, 40 ng for 0.05%, and 60 ng for 0.01% reference standards. The mutation allele fraction of NGS below 1% was filtered out. The results showed that the mutations can be enriched up to 3,600-fold. For example, cfDNA standards of EGFR 19Del at 0.1, 0.05, and 0.01% were enriched to ~75, ~65, and ~36% by PEAC, respectively. *EGFR* L858R mutation in the cfDNA standards at 0.1, 0.05, and 0.01% were amplified to ~61, ~45, and ~25% after PEAC enrichment (Figure 6). Overall, PEAC can achieve an average of 2,000-fold enrichment for mutations at 0.01% allele fraction, further demonstrating PEAC's ability to detect mutations with ultralow allele fractions followed by NGS.

## Discussion

In this study, we developed an ultrasensitive and cost-effective mutation detection system using plasma specimen. We improved BDA technologies by LNA modifications and shortening length in some nucleic acid probes, thus constructing the PEAC system to simultaneously detect 37 mutations in cfDNA. The false positive of BDA was solved by optimizing the length of blocker sequence and adding LNA modification on the blocker. As You et al. reported that shorter DNA probes which both bind to wildtype and mutant sequences have stronger discrimination power (58), we adopted the similar strategy and greatly reduced the false positives.

The PEAC system demonstrated high detection sensitivity both on cfDNA reference standard and clinical samples from NSCLC patients. The high sensitivity of PEAC system is due to the differential binding ability between the optimized primers and blockers. We achieved an overall specificity of 99% for all the mutations in cfDNA reference standards and 100% sensitivity for all the mutations at 1, 0.1, and 0.05% VAFs. For 0.01% VAF samples, we used 60 ng as the input DNA amount which only contains 1.8 mutant copies theoretically. Under this circumstance, PEAC still achieved a specificity of 99% and a sensitivity of 87%. The false negatives were very likely because the theoretical 1.8 mutant copies were lost during the preparation of ctDNA samples. Comparing with the results in TRACERx study (46), which showed 46% detection sensitivity when the allele fraction ranges between 0.01 and 0.05%, our PEAC system demonstrated much higher detection sensitivity at the allele fraction of 0.01%. We demonstrated 100% concordance rate in 29 NSCLC patients using NGS, ddPCR, and PEAC system. It is worth noting that PEAC could simultaneously detect many driver mutations from limited DNA amount, whereas ddPCR did not provide such ability. PEAC also achieved a 41% concordance rate between the paired tissue and plasma samples from 61 early stage patients with NSCLC (51 stage IA/IB and 10 stage II), which is outperformed than previously reported results (6, 56, 57). Given the above, PEAC system is applicable in the routine molecular laboratories to perform high sensitive ctDNA assay. In addition to detection by Sanger, PEAC enriched fragments can be analyzed by NGS workflow. With the multiplexing capability of all the enriched mutations, PEAC technology combined with NGS workflow has the potential application to develop high sensitivity and high throughput NSCLC mutation detection system.

The high sensitivity of NSCLC driver mutation detections using cfDNA plasma samples support the utility of PEAC to guide optimal treatment selection in targeted therapy. We demonstrated that using a cohort of 59 patients with NSCLC. Among them, 22 patients received first-generation EGFR-TKI treatment and achieved an overall ORR rate of 59.09%. PEAC also exclusively detected sensitive mutations (EGFR L858R or 19DEL) in 6 patients who had allele fractions below 0.05%. Most importantly, four patients who received TKI treatment were assessed and three of them had partial response (PR). These results further demonstrated the PEAC's ability to detect very low allele fraction mutations and the patients could still benefit from TKI treatment, and thus help to guide optimal treatment selections.

Another promising and potential application of PEAC system is MRD detection for patients with NSCLC to monitor tumor recurrence after surgery using 8–10 ml plasma samples. Since most patients who underwent surgery were in the early stages of the disease, and the postoperative ctDNA level in blood samples was usually very low. It is very important to improve the MRD detection rate for such circumstances, and PEAC enables high enough sensitivity at the single locus level. In addition, a convenient and cost-effective technology is required in the repeated ctDNA testing. Here, we used PEAC system to examine sequential blood samples from an NSCLC patient who was diagnosed with no recurrence by traditional examinations and serum tumor marker tests. Strikingly, PEAC identified EGFR L861Q mutation 6 months after surgery, which was previously detected in the tumor tissue sample, and 2 months later the patient was found tumor relapsed by MR examination. The presented case demonstrated that PEAC system could be a powerful tool for MRD monitoring. PEAC technology in this study enabled the enrichment and detection of 37 NSCLC driver mutations simultaneously using plasma sample. In principle, the technology is also applicable to enrich driver mutations and detect MRD for other cancer types as well. NGS-based methods for analyzing cfDNA need very high sequencing depth and complicated data analysis, and thus are less efficient and cost-effective methods when applied repeatedly. Besides, due to the tumor clone and subclone evolution, the low-multiplex PCR-based methods, which usually can only detect 2–3 hotspot mutations, are insufficient in clinical MRD detection (6, 7).

Although PEAC system has very high detection sensitivity and relatively large multiplexing capacity to detect the majority of NSCLC driver mutations using plasma samples, it has some limitations such as absolute variant allele fraction, which cannot be obtained currently due to the two-round PCR amplifications. So, by now it is not applicable to the applications which need accurate ctDNA quantification. Meanwhile, it does not limit its utilization in MRD detection and many clinical applications which only need to know the mutation status of the interested genomic loci. Another limitation is that the patient cohorts used to evaluate the performance of PEAC were retrospectively collected. To comprehensively investigate PEAC performance and its clinical benefits in MRD monitoring and early detection of cancers, we will need a prospective clinical trial to further validate the PEAC system.

## Conclusion

We have developed an ultrasensitive, large multiplexing capacity, and cost-effective NSCLC driver mutation detection system using ctDNA liquid biopsy. We validated PEAC by reference standard cfDNA samples and three cohorts of NSCLC patient samples. The results showed PEAC had very high detection sensitivity at the allele fractions below 0.05% and achieved quite consistent results on clinical samples. Those features make PEAC quite appropriate for emerging clinical applications, such as optimal selection of target therapies, early detection of cancers, and MRD monitoring for postsurgical patients using plasma samples.

## Data Availability Statement

The datasets presented in this study can be found in online repositories. The names of the repository and accession number can be found below: https://ngdc.cncb.ac.cn/gsa-human/browse/HRA002047, with accession no: HRA002047.

## Ethics Statement

The studies involving human participants were reviewed and approved by Ethics Committee of the Peking Union Medical College Hospital. The patients/participants provided their written informed consent to participate in this study.

## Author Contributions

TW and JX conceived the study. JX, TW, YP, and RL designed the experiment system. YP performed the experiments. JX provides clinical samples. JX, YP, SX, YF, TW, and RL interpreted the results. JX, YP, YF, RL, and TW wrote and edited the manuscript. All authors contributed to the article and approved the submitted version.

## Conflict of Interest

YP, RL, SX, YF, and TW are employees of Hangzhou Repugene Technology. The remaining author declares that the research was conducted in the absence of any commercial or financial relationships that could be construed as a potential conflict of interest.

## Publisher's Note

All claims expressed in this article are solely those of the authors and do not necessarily represent those of their affiliated organizations, or those of the publisher, the editors and the reviewers. Any product that may be evaluated in this article, or claim that may be made by its manufacturer, is not guaranteed or endorsed by the publisher.

## Supplementary Material

Supplementary Figure 1 Application of PEAC enrichment followed by Sanger sequencing on EGFR 19DEL, NRAS Q61K, and BRAF V600E.

Supplementary Figure 2 Application of PEAC enrichment followed by Sanger sequencing on EGFR G719S, S768I, and L861Q.

Supplementary Figure 3 Application of PEAC enrichment to closely located KRAS mutations.

Supplementary Figure 4 The adjustment of consistent sequencing depth among multiple mutations after PEAC enrichment.

Supplementary Table 1 The 37 mutations detected by PEAC system.

Supplementary Table 2 The sequences of forward primer-1, reverse primer-1, and the blocker.

Supplementary Table 3 The sequences of forward primer-2 and reverse primer-2.

Supplementary Table 4 The sequences of forward and reverse NGS primers, and their concentrations.

Supplementary Table 5 Overview of patient characteristics of three cohorts in this study.

Supplementary Table 6 Mutation profiles of the 29 lung cancer patients.

Supplementary Table 7 The mutation profiles of matched tissue and blood samples of the 76 NSCLC patients.
