# Supplementary figures and images for: PEAC: An Ultrasensitive and Cost-Effective MRD Detection System in Non-small Cell Lung Cancer Using Plasma Specimen
Source: Front Med (Lausanne). 2022 Mar 3;9:822200. doi: 10.3389/fmed.2022.822200 (PMC8928926; doi:10.3389/fmed.2022.822200)

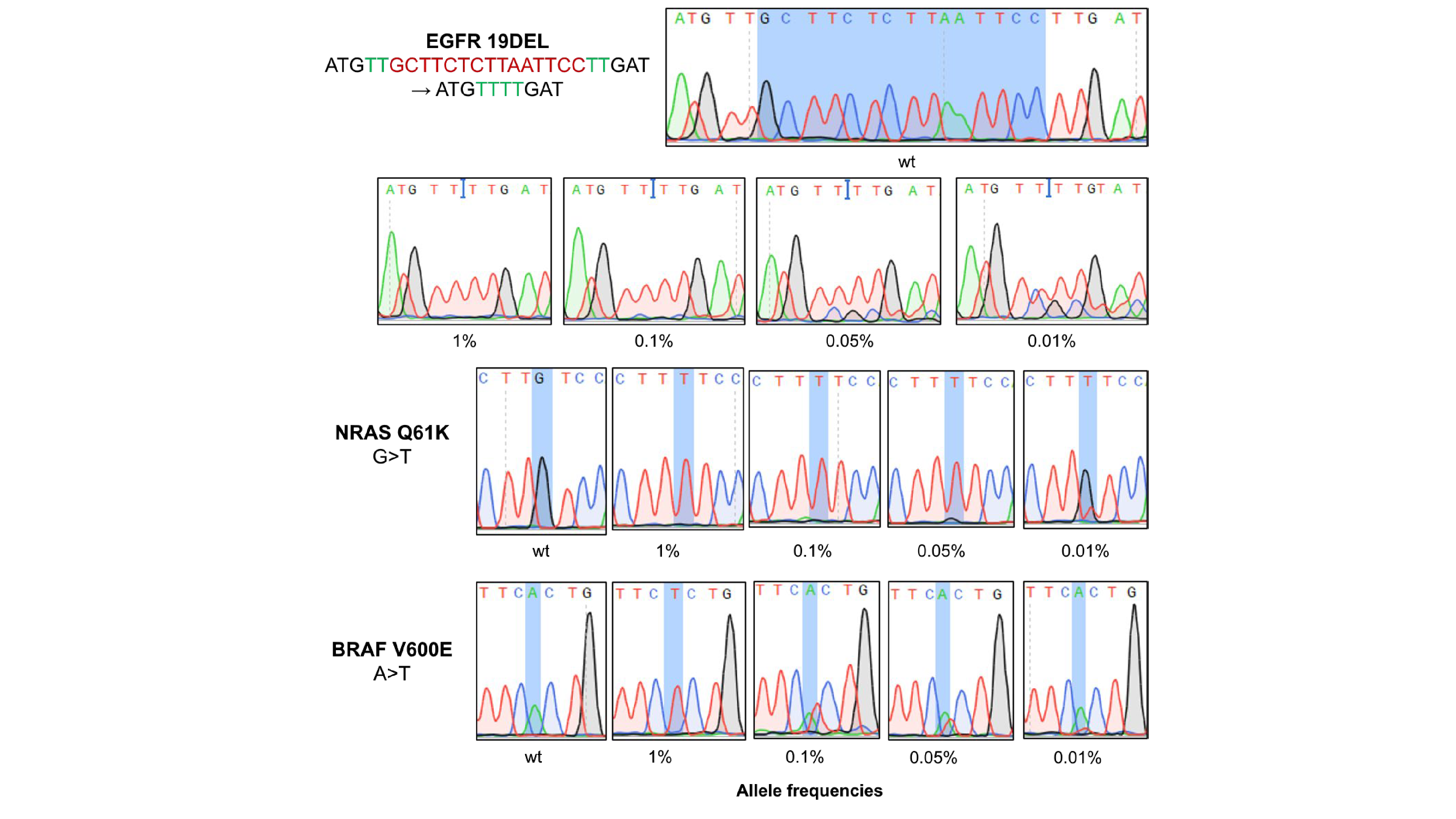

Supplement: Supplementary Figure 1 — Application of PEAC enrichment followed by Sanger sequencing on EGFR 19DEL, NRAS Q61K, and BRAF V600E. [file Image_1.TIF]

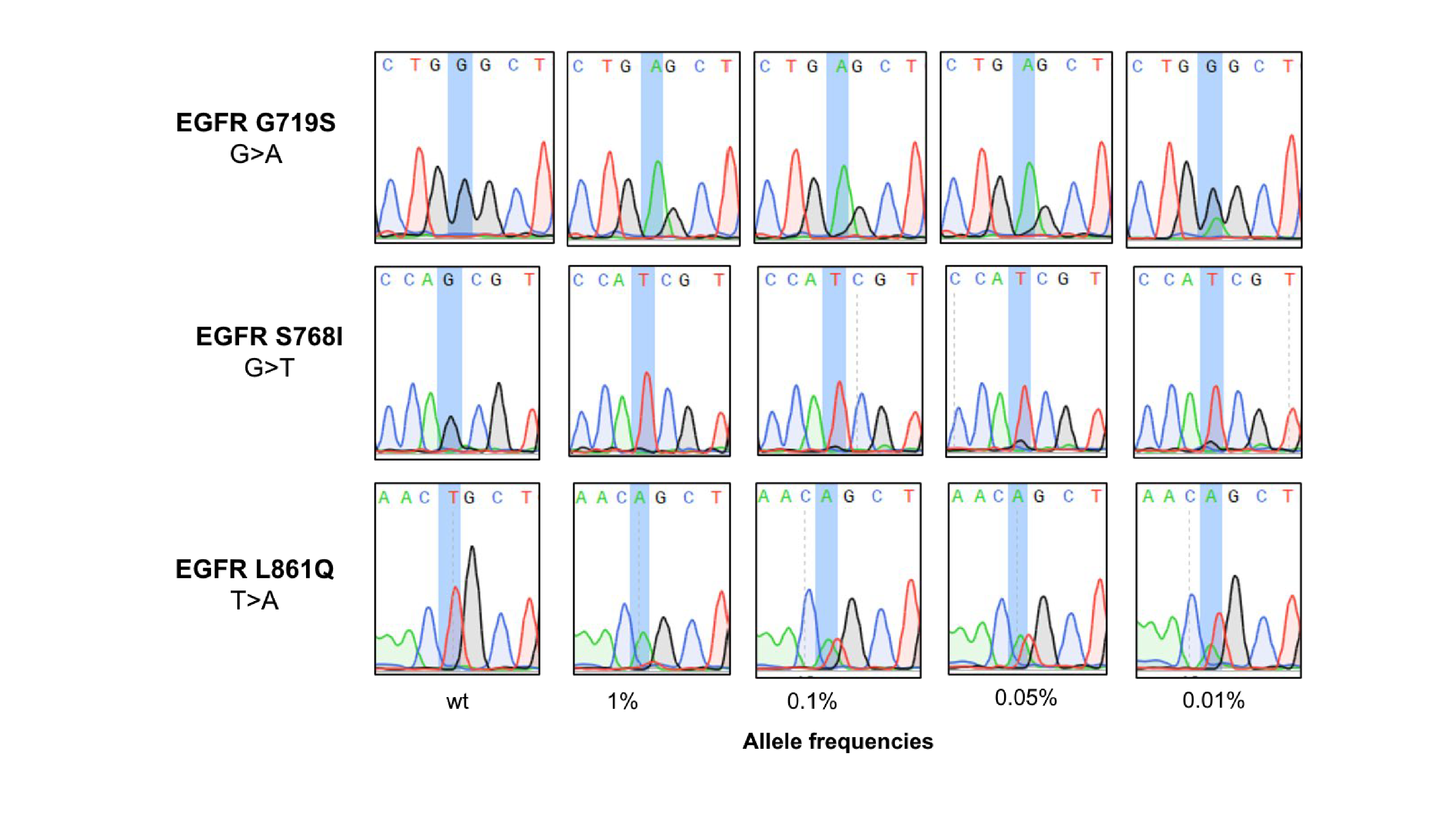

Supplement: Supplementary Figure 2 — Application of PEAC enrichment followed by Sanger sequencing on EGFR G719S, S768I, and L861Q. [file Image_2.TIF]

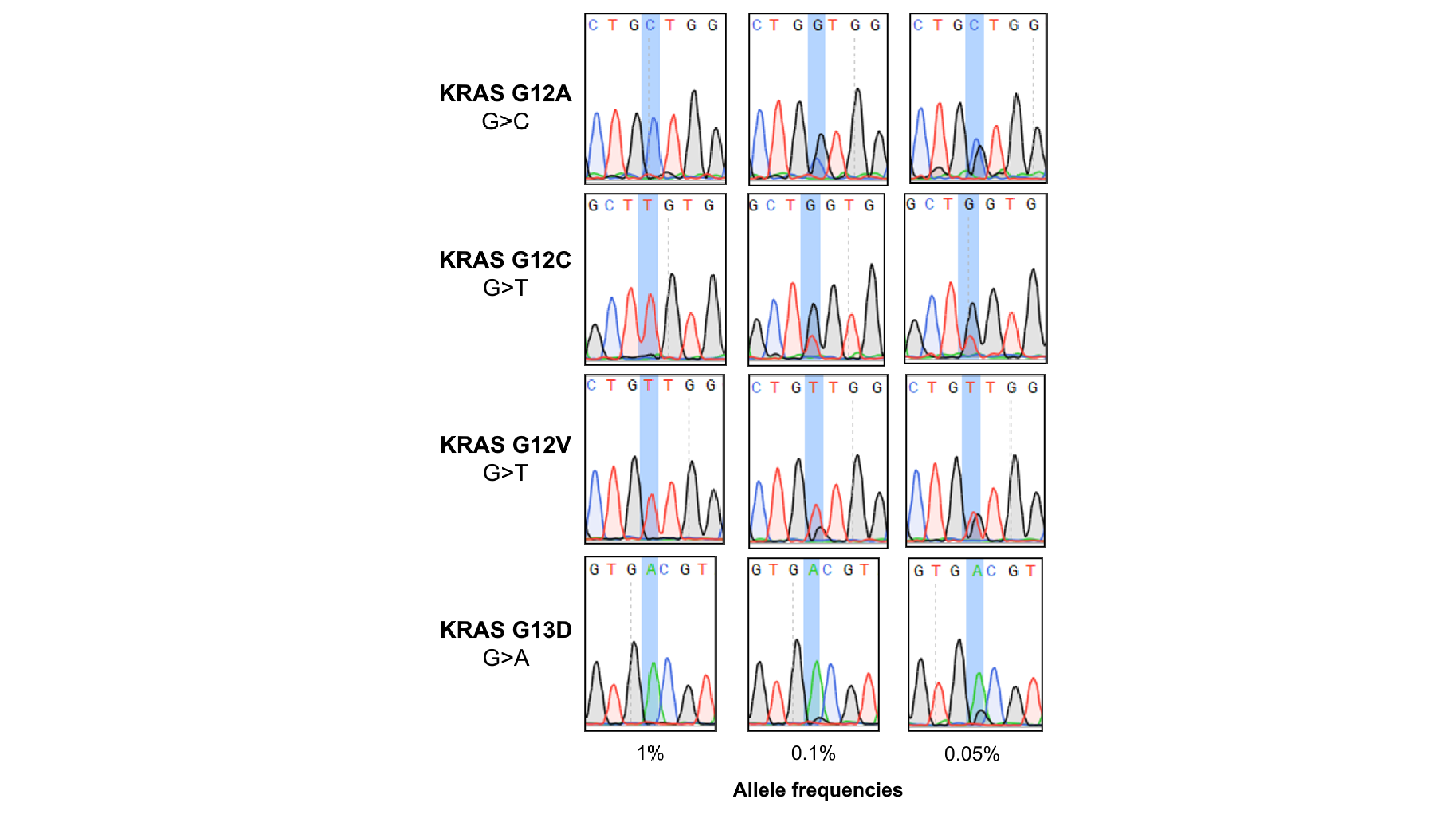

Supplement: Supplementary Figure 3 — Application of PEAC enrichment to closely located KRAS mutations. [file Image_3.TIF]

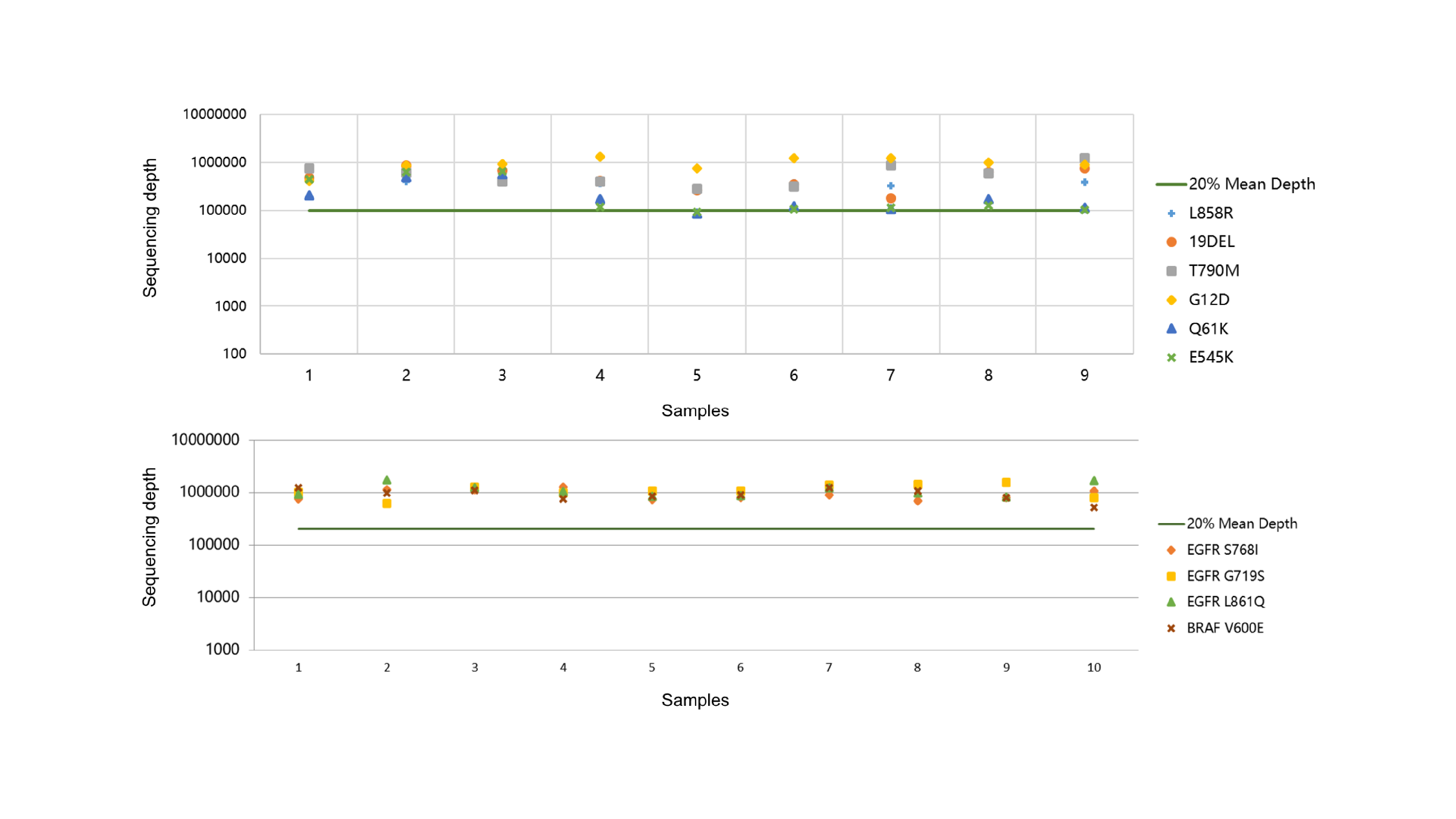

Supplement: Supplementary Figure 4 — The adjustment of consistent sequencing depth among multiple mutations after PEAC enrichment. [file Image_4.TIF]
